# Supplementary material for: The Construction and Exploration of a Comprehensive MicroRNA Centered Regulatory Network in Foxtail Millet (Setaria italica L.)
Source: Front Plant Sci. 2022 May 6;13:848474. doi: 10.3389/fpls.2022.848474 (PMC9121102; doi:10.3389/fpls.2022.848474)
Supplement: Supplementary file 4 [file Data_Sheet_4.zip › Sit-miR396b_Seita.9G141900.1_856_TPlot.pdf]

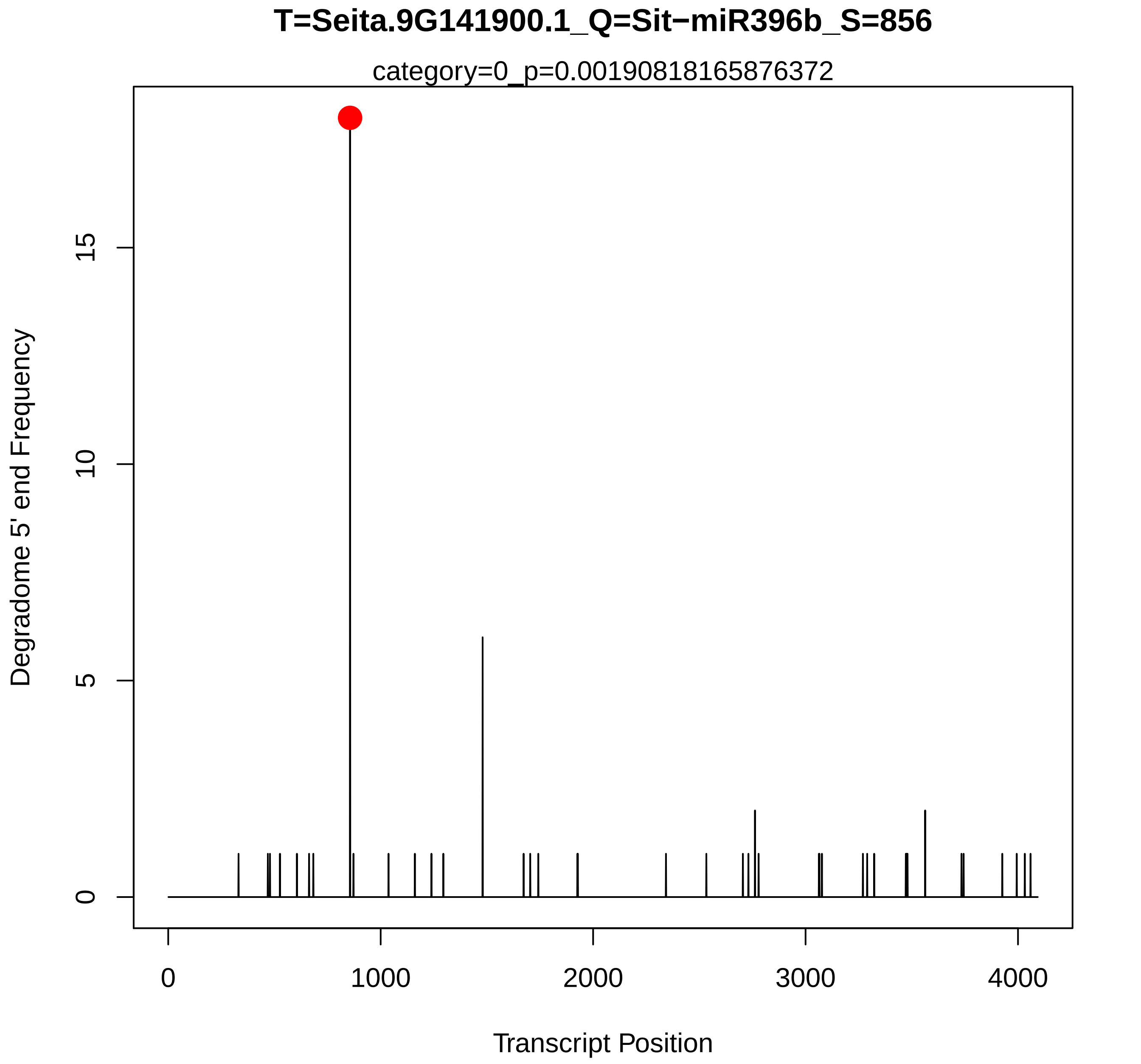

T=Seita.9G141900.1_Q=Sit−miR396b_S=856
category=0_p=0.00190818165876372
Degradome 5' end Frequency
0
5
10
15
0
1000
2000
3000
4000
Transcript Position
